# Health impact of providing informal care in Portugal

**DOI:** 10.1186/s12877-020-01841-z

**Published:** 2020-11-01

**Authors:** Fátima Barbosa, Gina Voss, Alice Delerue Matos

**Affiliations:** 1 Centro de Estudos de Comunicação e Sociedade, Instituto de Ciências Sociais, Universidade do Minho, Braga, Portugal; 2 Departamento de Sociologia, Instituto de Ciências Sociais, Universidade do Minho, Braga, Portugal

**Keywords:** Co-residential caregivers, Portugal, Physical health, Depressive symptoms, Longitudinal analysis, SHARE

## Abstract

**Background:**

Middle-aged and older adults play an important role in the provision of informal support, however, the impact on the health of those individuals who provide informal care is unclear. The main objectives of this study are: (1) to assess the prevalence of co-residential caregiving provided by individuals aged 50+; (2) to analyze differences between the group of Portuguese co-residential caregivers and the group of Portuguese non-caregivers; (3) to examine the longitudinal effect of providing informal care on the health of co-residential informal caregivers in Portugal.

**Methods:**

Data from wave 4 and wave 6 of the Survey of Health Ageing and Retirement in Europe (SHARE) were used. A linear mixed model and a generalized mixed model were used to analyze the longitudinal effect of providing informal care on the health (physical health and depressive symptoms) of Portuguese individuals aged 50 + .

**Results:**

In both SHARE waves analyzed, Portugal had the highest percentage of co-residential caregivers aged 50+. At baseline, the Portuguese co-residential caregiver population, compared to non-caregivers, has a lower percentage of employed individuals (14.9% compared to 25.7%) and a higher percentage of individuals with four or more depressive symptoms (56.4% compared to 35.5%). The caregivers also have a lower quality of life (CASP-12) (30.93 compared to 32.59). Marginal differences in educational levels between the caregiver and non-caregiver groups were also found, with co-residential caregivers having lower levels of education (72.3% have ISCED 0–2 compared to 64.7%), lower levels of cognitive function (− 2.321 compared to − 1.784), lower levels of physical health (− 0.180 compared to − 0.076) and lower engagement in moderate or vigorous physical activity (14.9% compared to 21.5%). Longitudinal models reveal that providing care within the household is not associated with physical health (b = 0.048; se = 0.035; *p* = 0.167), but *is* associated with depressive symptoms (OR = 1.609; 95% CI = 1.141–2.271; *p* = < 0.010).

**Conclusions:**

Portugal has the highest percentage of co-residential caregivers aged 50+. In that country, providing informal care to a household member is associated with depressive symptoms. Portuguese policymakers should therefore promote programs to prevent and alleviate the depressive symptoms experienced by individuals aged 50+, who provide co-residential care.

## Background

Middle-aged and older adults have assumed an important role in the provision of informal support [1]. They provide more informal support than they receive [2], and make a significant contribution to the health and social systems of their countries [1]. However, the impact of providing informal care on the health of these individuals is unclear [3]. If, on the one hand, longitudinal studies show that the provision of informal support has a negative impact on the physical and mental health of informal caregivers [4–6], on the other hand, similar studies show that informal caregivers have better levels of health, higher quality of life and lower levels of mortality compared to non-caregivers [7–10].

Despite these results, recent literature reviews have shown the negative health impact of providing informal care [11, 12]. A systematic review covering studies from various continents/countries (Europe, Asia, United States and Australia) highlights the negative impact of caregiving on the mental and physical health of the informal caregiver, especially female, married caregivers and those providing intensive care [11]. Cottagiri and Sykes [12] stress musculoskeletal disorders and psychological issues (such as depression, stress and anxiety) as the main health impacts of providing informal care.

Still on this issue, the literature highlights the need to distinguish between informal care provided inside the household and informal care provided outside the household, and between types of welfare state provision [13, 14]. Several studies have shown differences in physical and mental health between caregivers who provide informal care to a household member (co-residential care) and caregivers who provide care to a non-household member (extra-residential care) [13, 15–17]. Co-residential caregivers have greater physical and mental health problems. In contrast, extra-residential caregivers are more physically active and report better health [13, 15, 17]. On the subject of types of state welfare provision, the studies also show mixed results. Brenna and Di Novi [18], in their examination of the impact of providing constant care for elderly parents on the mental health of daughters aged 50–75 in different European countries, found a significant negative impact in Mediterranean countries only. The authors attribute this result to the fact that fewer resources are allocated to Long Term Care (LTC) and to the lack of social and health structures to meet the increasing demand for eldercare [18]. Comparing two European countries, Dujardin et al. [19] also concluded that, although caregiving was more prevalent in Britain, the health burden associated with heavy caregiving activities was lower in Britain than in Belgium. In contrast, Kaschowitz and Brandt [14] concluded that providing informal care inside the household results in a decline in mental health, irrespective of the type of welfare state.

Moreover, data from the U.S. Health and Retirement Study (2006, 2008, 2010, and 2012) demonstrate that spouse caregivers, new caregivers, continuing caregivers, and exit caregivers present elevated levels of depressive symptoms [7].

Despite the diversity of studies of this issue, the impact of providing informal care to a household member on the health of Portuguese caregivers aged 50+ has never been analyzed.

Considering that in Portugal the share of the population aged 65+ is expected to exceed one-third by 2050 [20] and the fact that Portugal has a lower public expenditure on LTC per person 65 years and over, and a lower percentage of the population aged 65+ receiving LTC at home or in an institution [21], this study is of the utmost importance for Portuguese policy makers and civil society. Moreover, over the last decades, Portugal has not consolidated formal Long Care Services as much as might be desired, and family is still the leading agent of care provision, as in the past [22].

Whereas informal caregiving is a growing public health issue [23] and given that previous studies indicate that Portugal is the country with the highest proportion of co-residential caregivers aged 50 plus in Europe, it is crucial to know the health impacts of providing care for people aged 50 years and older in Portugal. Therefore, the aims of this study are: (1) to assess the prevalence of co-residential caregiving provided by individuals aged 50+ by conducting a comparative analysis of European countries; (2) to analyze differences between the group of Portuguese co-residential caregivers and the group of Portuguese non-caregivers; (3) to examine the longitudinal effect of providing informal care on the health of co-residential informal caregivers in Portugal.

## Methods

### Study population

The current study uses data from the SHARE project (Survey of Health, Ageing and Retirement in Europe), an European multidisciplinary and cross-national panel database of micro data on health, socio-economic status and social and family networks [24]. A probability sample of the target population, i.e. individuals aged 50+, was interviewed in the SHARE project [24]. .Nevertheless, people who were incarcerated, hospitalized or out of the country during the entire survey period, or who were unable to speak the country’s language(s) or had moved to an unknown address were excluded from the survey. The interviewers used computer-assisted personal interviewing (CAPI) to collect the data. Proxy interviews were allowed when respondents were unable to do an interview, for example, for health reasons. For more methodological details of the SHARE project, please see Börsch-Supan et al. [24].

Due the fact that Portugal only started participating in the SHARE project in wave 4 (2011) and did not participate in wave 5 (2013), the current study uses data from wave 4 (2011) and wave 6 (2015) (release 6.1.0.).

We restricted our sample to SHARE Portuguese respondents aged 50+ who participated in waves 4 and 6, and who did not have missing information for question “*Is there someone living in this household whom you have helped regularly during the last twelve months with personal care, such as washing, getting out of bed, or dressing?”* in both the waves analyzed (4 and 6) (*N* = 1262).

### Measures

#### Outcome variables

Health was examined by two measures: physical health and depressive symptoms.

Physical health was assessed using a latent continuous measure. This variable was created according to procedures in Ploubidis and Grundy [25] and Di Gessa et al. [26] and includes one objective health indicator (maximum grip strength, using one or both hands) and six subjective ones. The subjective variables used are: self-perceived health (*Would you say your health is …*) using a 5-point ordinal scale (poor (1), fair (2), good (3), very good (4) or excellent (5)); the presence of long-term illness (*Some people suffer from chronic or long-term health problems. By chronic or long-term we mean it has troubled you over a period of time or is likely to affect you over a period of time. Do you have any such health problems, illness, disability or infirmity?)*: coding 0 if yes and 1 if no; limited activities because of health (*For the past 6 months at least, to what extent have you been limited in your activities because of a health problem*): coding 1 for severely limited; 2 for limited, but not severely; and 3 for not limited; the doctor told that you have or had a heart attack: coding 0 for yes, and 1 for no; the doctor told that you have or had a stroke: coding 0 for yes, and 1 for no; and the doctor told that you have or had a chronic lung disease: coding 0 for yes, and 1 for no. This physical health measure was implemented in MPLUS, version 7, using WLSMV estimator (Muthén & Muthén, 1998–2012).

According to Ploubidis and Grundy [25], this measure is less subject to measurement error and has greater repeatability and reliability compared to individual health indicators used separately. In both waves (waves 4 and 6), our model revealed a good model fit: the Root Mean Square Error of Approximation (RMSEA) was 0.030 in wave 4 and 0.033 in wave 6 (values less than 0.06 indicate good fit); the Comparative Fit Index (CFI) was 0.986 in wave 4 and 0.983 wave 6 and the Tucker-Lewis Index (TLI) was 0.979 in wave 4 and 0.974 in wave 6 (for adequate models, both indices should have values above 0.95).

Depressive symptoms were assessed using the EURO-D 12-item scale that includes questions about feelings of depression, pessimism, wishing death, guilt, irritability, tearfulness, fatigue, sleeping troubles, loss of interest, loss of appetite, reduction in concentration, and loss of enjoyment over the last month [27]. Each question was scored one, if the feeling was present, or zero, if the feeling was not present, with a minimum possible score of zero and a maximum possible score of twelve.

We follow the Dewey and Prince [28] procedures and define clinically significant depressive symptoms as a EURO-D score greater than 3; and no clinically significant depressive symptoms as a EURO-D score equal or lower than 3. According to the same authors [28], this cutpoint was validated in the EURODEP study across the continent, and against a variety of clinically-relevant indicators. A EURO-D score greater than 3 would be likely to be diagnosed as suffering from a depressive disorder, for which therapeutic intervention would be indicated [28]. Psychometric evaluation of the 12 individual scale items for Portugal revealed good Cronbach’s Alpha in wave 4 (0.82) and acceptable in wave 6 (0.76) [22].

#### Independent variable

In the current study, informal care was defined as non-professional, unpaid support given to a family member, friend, neighbour or someone with another type of relationship living inside or outside their household who requires help with everyday tasks [14, 29]. The provision of informal care inside the household was analyzed by question: *Is there someone living in this household whom you have helped regularly during the last 12 months with personal care, such as washing, getting out of bed, or dressing?* In this question, SHARE considers that regularly means daily or almost daily informal care provided for at least 3 months. Taking this question into consideration, we defined as an informal caregiver inside the household all the Portuguese aged 50+ who responded affirmatively.

#### Covariates

Based on the literature, the current analysis included several control variables.

Age at the time of interview, sex (1 = female and 0 = male), marital status (1 = married and living together and 0 = all the other situations) and current job situation (1 for employed and 0 for all the other categories: retired, unemployed, permanently sick, homemaker and other). Education was coded according to the International Standard Classification of Education 1997 (ISCED-97). Respondents were grouped into the following categories: 1 as primary education (ISCED-97 score = 0–2), 2 as secondary education (ISCED-97 score = 3), and 3 as post-secondary education (ISCED-97 score = 4–6) [30]. Income was constructed using the variable total household net income (version A) that is obtained by a suitable aggregation at the household level of all individual income components. Income was adjusted for purchasing power parity and the square root of household size and divided into tertiles. The lowest tertile was coded as 1, the middle as 2, and the highest as 3. Cognitive function was constructed according to the procedures in Leist et al. [31]. The sum of five z-score measures was used: immediate recall (immediately recalling as many words as possible from a 10-word list that had been read out); delayed recall (recalling the ten-word list after a short delay); numeracy (assessed by five arithmetical subtraction tasks); Verbal fluency (naming as many animals as possible in 1 min) and orientation (score of orientation in time test). For the construction of this variable, we only consider individuals who had valid values for at least three of the tests.

To assess physical inactivity, SHARE respondents were asked how often they engage in vigorous activity (i.e., sport, heavy housework, or a job that requires physical labour) or moderate activity (i.e., activities requiring a low or moderate level of energy such as gardening, cleaning the car, or walking), with four response options: 1 - more than once a week; 2 - once a week; 3 - one to three times a month; 4 - hardly ever or never. In this study, we used the generated dummy variable that characterizes physically inactive individuals as those who have never practised vigorous or moderate physical activity.

Social network scale was a summary scale that combines five social network characteristics within a single index [30]. These characteristics include (1) the number of persons cited (network size); (2) the number of cited social network members living within 25 km (proximity); (3) the number of cited persons with weekly or more contact (frequency); (4) the number of cited persons with very or extremely close emotional ties (support); and (5) the number of different types of relationships present within the network (diversity). The first four measures were scored as follows: 0 = 0 SN members; 1 = 1 SN member; 2 = 2–3 SN members; 3 = 4–5 SN members; 4 = 6–7 SN members. The fifth measure calculated the number of different relationship categories (1- spouse; 2- other family, including children; 3- friend; and 4- other) present in the network. This last measure score ranged from 0 to 4, with a score of zero meaning no social network (i.e. no persons named) and the remainder reflecting the number of different relationship types, from 1 to 4. The total social network scale varies between 0 and 20, with 0 representing no named people in the social network and higher scores representing more social capital. This scale was divided into five levels (0 to 4), with the lower level (0) representing no social network (i.e. no people named), level 1 representing scores 1 to 5, level 2 scores 6 to 10, level 3 scores 11 to 15 and level 4 scores 16 to 20. Psychometric evaluation of the five individual scale items for Portugal revealed high Cronbach’s Alpha in both waves analyzed (0.93 in wave 4 and 0.81 in wave 6).

Finally, Quality of Life (QoL) was assessed using the CASP-12 scale, the short version of CASP-19 [32], which comprises four dimensions: Control, Autonomy, Self-realization and Pleasure. The total number of points on the CASP-12 scale varies between 12 and 48 points, with a greater QoL corresponding to higher values. Cronbach’s Alpha in wave 4 was 0.83 and in wave 6 it was 0.50.

### Statistical analysis

Firstly, we assessed the prevalence of informal caregiving inside the household provided by individuals aged 50+, comparing Portugal with other SHARE countries. Secondly, we compared baseline characteristics (wave 4, 2011) of Portuguese individuals aged 50+ who provided informal care inside the household (co-residential caregivers) with the characteristics of those who do not provide informal care inside the household (non-caregivers). Statistical tests (chi-square test and T test) for two-group comparison were applied. Statistical test results with *p* < 0.05 were considered to be significant, and with *p* < 0.10 were considered marginally significant. Thirdly, a longitudinal linear mixed model with fixed effects, random effects and an error was used to analyze the impact of providing informal care inside the household on the physical health of Portuguese individuals aged 50+. The model was fitting by maximum likelihood, using the following equation: ***Y***_***ij***_ ***= C***_***ij***_***β + X***_***ij***_***δ + U***_***i***_ ***+ Z***_***ij***_, ***i =*** **1*,*** …***,n;j =*** **1,2** , where, ***Y***_***ij***_ denotes the dependent variable – physical health – for the individual ***i*** at time ***j***, ***C***_***ij***_ is a dummy for caregiver inside the household or not, ***X***_***ij***_ is a vector that includes all control variables, the ***U***_***i***_ is the individual random effect, ***U***_***i***_***~N***(**0**, ***ν***^**2**^), and ***Z***_***ij***_ is the measure error, ***Z***_***ij***_***~N***(**0**, ***τ***^**2**^), with ***U***_***i***_ and ***Z***_***ij***_ being independents. Thus, ***β*** gives the effect of providing care inside the household (vs. not providing care inside the household) for the individual ***i*** [33]. Lastly, a longitudinal generalized mixed model with a logit link function was applied to analyze the impact of providing care inside the household on the depressive symptoms of Portuguese individuals aged 50+. To this end, we consider constant correlation and the equation.

$$ \boldsymbol{\ln}\left(\frac{{\boldsymbol{\pi}}_{\boldsymbol{i}\boldsymbol{j}}}{\mathbf{1}-{\boldsymbol{\pi}}_{\boldsymbol{i}\boldsymbol{j}}}\right)={\boldsymbol{C}}_{\boldsymbol{i}\boldsymbol{j}}\boldsymbol{\beta} +{\boldsymbol{X}}_{\boldsymbol{i}\boldsymbol{j}}\boldsymbol{\delta} +{\boldsymbol{U}}_{\boldsymbol{i}}+{\boldsymbol{Z}}_{\boldsymbol{i}\boldsymbol{j}},\kern0.5em \boldsymbol{i}=\mathbf{1},\dots, \boldsymbol{n};\boldsymbol{j}=\mathbf{1},\mathbf{2}, $$

where ***π***_***ij***_ denotes the probability of success (being depressed) for the individual ***i*** at time ***j*** (***P***(***Y***_***ij***_ ***=*** **1**)), and the $\left(\frac{{\boldsymbol{\pi}}_{\boldsymbol{ij}}}{\mathbf{1}-{\boldsymbol{\pi}}_{\boldsymbol{ij}}}\right)$ is called the odd ratio (***OR***). The ***C***_***ij***_ is a dummy variable for providing care inside the household (vs. not providing care inside the household), ***X***_***ij***_ is a vector that includes all control variables, the ***U***_***i***_ is the individual random effect, ***U***_***i***_***~N***(**0**, ***ν***^**2**^), and ***Z***_***ij***_ is the measurement error, ***Z***_***ij***_***~N***(**0**, ***τ***^**2**^), being ***U***_***i***_ and ***Z***_***ij***_ independents. In this way, **exp**(***β***) gives the likelihood of a caregiver inside the household being depressed over a non-caregiver for the individual ***i***. All analyses were performed using software R, version 3.4.3.

## Results

**Fig. 1:**
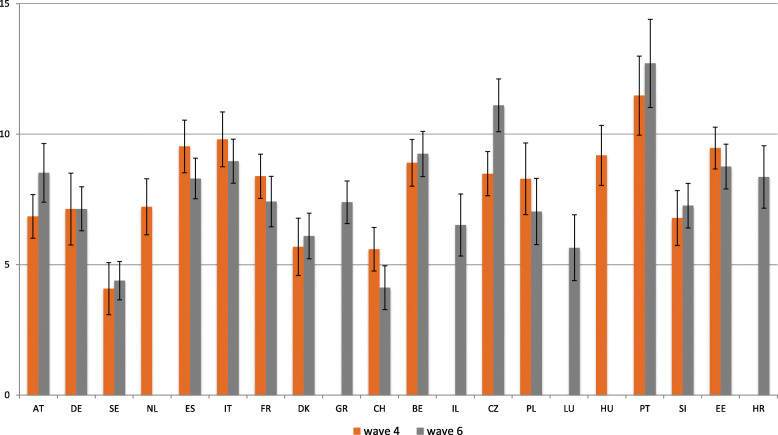
Prevalence of informal caregiving, according to country and wave (wave 4 and 6). Source: SHARE Wave 4 and 6 release 6.1.0.; weighted data; N of co-residential caregivers (all countries) in wave 4 = 3796 and wave 6 = 4494. Note: Brackets denote a 95% confidence interval

Figure 1 shows the percentage of individuals aged 50+ who provide informal care inside the household, by country and wave (wave 4 and 6). It reveals that, in wave 4, Portugal (11.5%), Italy (9.8%), Spain (9.5%), Estonia (9.5%) and Hungary (9.2%) were the countries with the highest percentage of co-residential caregivers aged 50+. Whereas in wave 6, Portugal (12.7%), Czech Republic (11.1%), Belgium (9.2%) and Italy (9%), are the countries that show the highest percentages of this kind of support. In both waves analyzed; the highest percentage of co-residential caregivers aged 50+ is found in Portugal.

By contrast, Sweden (wave 4: 4.1% and wave 6: 4.4%) and Switzerland (wave 4: 5.6% and wave 6: 4.1%) are the countries with the lowest percentages of co-residential caregivers aged 50 + .

Taking into consideration the Portuguese sample only, we perform descriptive analyses for 141 Portuguese co-residential caregivers and 1121 non-caregivers (Table 1). At baseline wave (wave 4, 2011), co-residential caregivers differ significantly (*p* = < 0.05) from non-caregivers in terms of their current job situation (*p* = 0.005), depressive symptoms (*p* = < 0.001) and quality of life (*p* = < 0.001), and differ marginally (*p* = < 0.10) in terms of education (*p* = 0.080), cognitive function (*p* = 0.065), physical health (*p* = 0.075) and physical inactivity(*p* = 0.069) (Table 1). The Portuguese co-residential caregiver group compared with the non-caregiver group have a lower percentage of employed individuals (14.9% compared to 25.7% in the non-caregiver group), have a higher percentage of individuals with four or more depressive symptoms (56.4% compared to 35.5% in non-caregiver group) and have a lower quality of life (CASP-12) (30.93 compared to 32.59 in the non-caregiving population). With regard to the marginal differences between groups, the co-residential caregiver group has lower levels of education (72.3% have ISCED 0–2 compared to 64.7% in the non-caregiver group), lower levels of cognitive function (− 2.321 compared to − 1.784 in non-caregivers group), lower levels of physical health (− 0.180 compared to − 0.076 in the non-caregiver group) and a lower percentage of individuals who are engaged in moderate or vigorous physical activity (14.9% compared to 21.5% in the non-caregiver group).

**Table 1:** Descriptive statistics of Portuguese individuals aged 50+ at baseline (wave 4)

	Individuals who provide caregiving inside the household	Individuals who do not provide caregiving inside the household	***p*** value	t/x^**2**^
	(***N*** = 141)	(***N*** = 1121)		
Age, mean (SD)	63.26 (8.57)	64.40 (8.94)	0.135	−1.494
Sex			0.222	1.489
Male	40.4%	45.9%		
Female	59.6%	54.1%		
Current job situation			0.005	7.955
Employed	14.9%	25.7%		
Other situation	85.1%	74.3%		
Marital status			0.506	0.443
Married and living together	85.8%	87.8%		
Other situation	14.2%	12.2%		
Education (ISCED-97)			0.080	5.045
Isced-97 (0–2)	72.3%	64.7%		
Isced-97 (3)	2.1%	6.0%		
Isced-97 (4–6)	25.5%	29.3%		
Income			0.684	0.761
Tertile 1	36.2%	33.6%		
Tertile 2	36.1%	35.2%		
Tertile 3	27.7%	31.1%		
Depressive symptoms (4 or more)			< 0.001	23.023
No	43.6%	64.5%		
Yes	56.4%	35.5%		
Cognitive function, mean (SD)	−2.321 (3.141)	−1.784 (3.261)	0.065	1.850
Physical health, mean (SD)	−0.180 (0.630)	−0.076 (0.632)	0.075	1.781
Physical inactivity			0.069	3.309
Active	14.9%	21.5%		
Inactive	85.1%	78.5%		
Social network scale (0–4), mean (SD)	2.34 (1.415)	2.24 (1.354)	0.415	0.816
Quality of Life (CASP-12), mean, (SD)	30.93 (5.016)	32.59 (4.862)	< 0.001	3.708

Source: SHARE Wave 4 release 6.1.0.; unweighted data; *P* values refer to the relevant statistical tests for two-group comparison (i.e. T test for independent samples (t); chi-square tests (X^2^))

Descriptive analyses also reveal that 26% [34] of Portuguese co-residential caregivers of wave 4 were still providing informal care inside the household in wave 6.

Table 2 shows that, after controlling for all confounders, providing care inside the household does not have a significant effect on the physical health (b = 0.048; se = 0.035; *p* = 0.167) of Portuguese individuals aged 50+. On the contrary, caregivers are 60.92% more likely to report four or more depressive symptoms than non-caregivers (OR = 1.609; 95% CI = 1.141–2.271; *p* = < 0.010).

**Table 2:** Regression results for caregiving inside the household and health

Physical health				Depressive symptoms		
	b	se	***p*** value	OR	IC (95%)	***p*** value
Caregiving inside the household	0.048	0.035	0.167	1.609	1.141–2.271	0.007
Observations/persons	2124/1213			2124/1213		

Note: SHARE Wave 4 and wave 6, release 6.1.0.; *b* Coefficients; *se* Standard error, *OR* Odds Ratio, *IC* Intervals Confidence, *Pr* Probability, *I* Controls: age, sex, current job situation, marital status, education, income, depressive symptoms, cognitive function, physical inactivity, social network scale and quality of life; II = Controls: age, sex, current job situation, marital status, education, income, physical health, cognitive function, physical inactivity, social network scale and quality of life; own calculations, unweighted. *p < .05, **p < .01, ***p < .001

## Discussion

Due to the increasing demand for informal support, the number of middle-aged and older adults who are providing informal care is rising [4]. According to our results, Portugal has the highest percentage of co-residential caregivers aged 50 plus in both waves analyzed (waves 4 and 6). These results are in line with several studies highlighting that co-residential care is more prevalent in Southern European countries [13, 34, 35], which are characterized as familistic [36] and where the responsibility for long-term care (LTC) is mainly assumed by families [37].

The descriptive analysis, at baseline (wave 4), also shows important differences between Portuguese co-residential caregivers and their non-caregiver counterparts, namely in terms of employment status, education, health and quality of life. These findings are consistent with other cross-sectional studies indicating that co-residential caregivers are less likely to be employed [16]. Considering that co-residential care is associated with more intensive care and the fact that Portugal has less generous formal long-term care provision [34], this activity may prevent reconciliation between caregiving and employment. Regarding the caregiver’s educational level, our results reinforce previous findings [16, 17], which show that co-residential care is associated with lower educational levels. Furthermore, less educated caregivers also receive lower levels of paid support [38], which can increase the intensive care and number of hours of care provided. Portuguese co-residential caregivers also report worse physical health [14, 16], a higher percentage of depressive symptoms [15], as well as lower quality of life [13]. In Portugal, these characteristics may be related to the higher level of care provided. As co-residential caregiving is significantly associated with more hours of care and more chronic stress, co-residential caregivers may experience physical and emotional exhaustion, as well as worse self-perceptions of physical and mental health [14, 39, 40]. With regard to these results, Kaschowitz and Brandt [14] explained that there is selection into caregiving, with people in worse health tending to choose to provide care inside the household while people in better health take up care outside the household. According to the same authors, people in worse health have fewer opportunities for work and a higher likelihood of assuming the role of informal caregiver at home [14].

In relation to cognitive function and physical inactivity, our results do not corroborate the recent literature that points to the healthy caregiver hypothesis. In fact, Portuguese co-residential caregivers show lower cognitive function and less physical activity compared to non-caregivers [41].

Despite the fact that Portuguese co-residential caregivers differ from non-caregivers in terms of physical health and depressive symptoms at baseline, longitudinal analysis shows that providing informal care inside the household only has a detrimental effect on depressive symptoms. These results corroborate the findings of Roth et al. [3]. These authors argued that, in well-controlled population-based studies, there is very little evidence that family caregivers have poor objective physical health compared to non-caregivers. In relation to depressive symptoms, our results are in line with the literature [4, 14], which shows that providing co-residential care has a significant negative impact on the mental health of middle-aged and older individuals. Sharing the same environment with the person receiving care, the high number of hours spent giving care, the higher levels of neuroticism and the emotional distress of continuous exposure to the suffering of a loved one [3, 14, 42, 43] are important factors that can explain this effect. Moreover, the subjective caregiver burden is pointed out as a significant risk factor for depressive symptoms that can lead to clinical depression [44]. Overall, our results are in line with previous studies that showed that providing co-residential care does not have a negative impact on objective physical health [3], but only on mental health [6, 14].

According to our research, middle-aged and older Portuguese who are providing this type of care face important challenges in terms of depressive symptoms. The existence of modest LTC services [29] and the unequal distribution of mental health services in Portugal [45] may jeopardize the mental health of Portuguese co-residential caregivers. In this sense, it is crucial to reconfigure and consolidate Portuguese public social and health care services to promote greater equity in access to health and social services. Therefore, considering the expected growing number of informal caregivers in the coming years, Portuguese policy makers should formally recognize informal caregivers and provide them with multidisciplinary services capable of preventing and alleviating the detrimental health effects of providing informal care. More practical research should also be conducted to better understand the real challenges faced by Portuguese informal caregivers as well as their needs and to create support systems capable of promoting the mental health of informal caregivers.

This study has strengths and limitations. To our knowledge, this is the first-ever study to analyze the health impact of providing informal co-residential care on Portuguese individuals aged 50+ and to analyze physical health as a latent continuous physical health measure that combines subjective and objective indicators. Nevertheless, this study has some limitations. Firstly, the low number of respondents did not allow us to perform an analysis by caregiver relation type (spouse, child, friend, etc.). Secondly, the SHARE project did not ask the number of hours of care provided, and therefore we are not able to analyze care intensity.

## Conclusions

This is the first-ever study to analyze the impact of providing co-residential informal care on the health of Portuguese individuals aged 50+. The current study shows that Portugal is the country with the highest percentage of co-residential caregivers aged 50+ and that providing co-residential care in Portugal is associated with having four or more depressive symptoms. These findings have important implications for Portuguese public policies. In view of the rapidly ageing Portuguese population [29], and the fact that the highest percentage of co-residential caregivers is in the 50+ population, Portuguese public policies will face great challenges in the near future.

## Data Availability

This paper uses data from SHARE Waves 4 and 6 release 6.1.0 as of 28 March 2018 (DOIs: 10.6103/SHARE.w4.610 and 10.6103/SHARE.w6.610). The SHARE data are available and can be downloaded from the SHARE Research Data Center under the following conditions: Applicants must have a scientific affiliation and have to sign a statement confirming that under no circumstances will the data be used for other than purely scientific purposes. Data will only be made available after these documents have been received, by mail or fax (care of Josette Janssen; address: CentERdata, Tilburg University, P.O. Box 90153, 5000 LE Tilburg, The Netherlands; e-mail: jjanssen@uvt.nl. Methodological details of the SHARE study design and data collection are presented elsewhere (Börsch-Supan et al., 2013).

## Abbreviations

SHARE: Survey of Health, Ageing and Retirement in Europe
LTC: Long Term Care
RMSEA: Root Mean Square Error of Approximation
CFI: Comparative Fit Index
TLI: Tucker-Lewis Index
ISCED-97: International Standard Classification of Education-97
OECD: Organisation for Economic Co-operation and Development
CI: Confidence interval
OR: Odds Ratio
